# 426. Effectiveness of Updated (2023-2024) Monovalent COVID-19 Vaccines – VISION Network, September 2023-March 2024

**DOI:** 10.1093/ofid/ofae631.140

**Published:** 2025-01-29

**Authors:** Ruth Link-Gelles, Elizabeth Rowley, Sarah E Reese, Omobosola Akinsete, Kristin K Dascomb, Stephanie Irving, Nicola P Klein, Shaun J Grannis, Toan Ong, Sarah W Ball, Katherine E Fleming-Dutra, Malini DaSilva, Allison L Naleway, Ousseny Zerbo, Bruce Fireman, Brian E Dixon, Karthik Natarajan, Mark W Tenforde, Amanda B Payne

**Affiliations:** Centers for Disease Control and Prevention, Atlanta, Georgia; Westat, Durham, North Carolina; Westat, Durham, North Carolina; HealthPartners Institute, Minneapolis, Minnesota; Intermountain Healthcare, Murray, Utah; Kaiser Permanente Center for Health Research, Portland, Oregon; Division of Research Kaiser Permanente Vaccine Study Center, Oakland, California; Indiana University, Indianapolis, Indiana; University of Colorado Anschutz Medical Campus, Centennial, Colorado; Westat, Durham, North Carolina; Centers for Disease Control and Prevention, Atlanta, Georgia; HealthPartners Institute, Minneapolis, Minnesota; Kaiser Permanente Center for Health Research, Portland, Oregon; Division of Research Kaiser Permanente Vaccine Study Center, Oakland, California; Division of Research Kaiser Permanente Vaccine Study Center, Oakland, California; Regenstrief Institute, Indianapolis, Indiana; Columbia University, New York, New York; US Centers for Disease Control and Prevention, Decatur, Georgia; CDC, Atlanta, Georgia

## Abstract

**Background:**

In September 2023, the Centers for Disease Control and Prevention’s Advisory Committee on Immunization Practices (ACIP) recommended updated 2023-2024 (monovalent XBB.1.5) COVID-19 vaccines for use in persons ≥6 months for prevention of COVID-19, including severe disease. Understanding how well updated COVID-19 vaccines work in the context of high population immunity due to prior infection, vaccination, or both, is important for future vaccine policy decisions, as well as informing patient/provider discussions, and increasing vaccine confidence.

**Figure:**
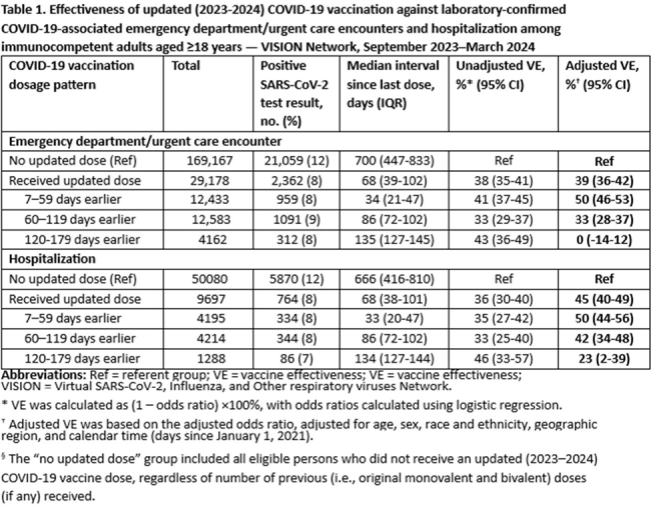

Effectiveness of updated (2023-2024) COVID-19 vaccination against laboratory-confirmed COVID-19-associated emergency department/urgent care encounters and hospitalization among immunocompetent adults aged ≥18 years — VISION Network, September 2023–March 2024

**Methods:**

VISION, an electronic health record (EHR)-based network including emergency departments/urgent care clinics (ED/UCs) and hospitals including 6 health systems, uses clinician-ordered testing data to estimate vaccine effectiveness (VE) for respiratory viruses. COVID-19 VE was estimated using the test-negative design, comparing the odds of vaccination with a single updated 2023-2024 COVID-19 vaccine dose between patients who tested positive versus those who tested negative for SARS-CoV-2 by molecular assay, adjusting for potential confounders.

**Figure:**
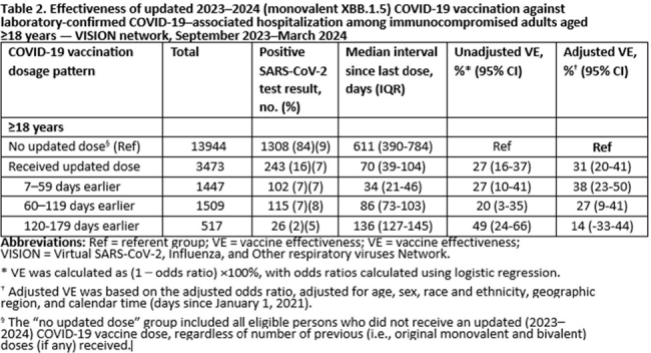

Effectiveness of updated 2023–2024 (monovalent XBB.1.5) COVID-19 vaccination against laboratory-confirmed COVID-19–associated hospitalization among immunocompromised adults aged ≥18 years — VISION network, September 2023–March 2024

**Results:**

A total of 198,345 ED/UC encounters and 59,777 hospitalizations between September 2023-March 2024 among immunocompetent adults ≥18 years with symptoms of COVID-19 were included. VE against COVID-19-associated ED/UC encounters comparing receipt of an updated dose to no receipt of an updated dose was 50% (95% CI: 46-53%) in the 7-59 days and 0 (95% CI: -14 to 12) in the 120-179 days after receipt of an updated dose (Table 1). VE against COVID-19-associated hospitalization was 50% (95% CI: 44-56%) in the 7-59 days and 23 (95% CI: 2-39) in the 120-179 days after receipt of an updated dose (Table 1). A total of 17,417 hospitalizations among immunocompromised adults ≥18 years with symptoms of COVID-19 were included, with a VE of 38% (95% CI: 23-50%) in the 7-59 days and 14 (95% CI: -33 to 44) in the 120-179 days after receipt of an updated dose after an updated dose (Table 2).

**Conclusion:**

Receipt of an updated COVID-19 vaccine dose provided protection against COVID-19-associated ED/UC encounters and hospitalizations among immunocompetent and immunocompromised adults, although waning was evident.

**Disclosures:**

**Nicola P. Klein, MD, PhD**, GlaxoSmithKline: Grant/Research Support|Merck: Grant/Research Support|Pfizer: Grant/Research Support|Sanofi Pasteur: Grant/Research Support|Seqirus: Grant/Research Support **Toan Ong, PhD**, Patent Title: Systems and Methods For Record Linkage: PCT/US2018/047961 Patent Title: Systems and Methods For Record Linkage|PCORI: Travel support to attend the PCORI Annual meeting in Washington DC, 2023|Regenstrief Institute: Advisor/Consultant|Regenstrief Institute: Travel support to attend the OHIE 23 meeting in Malawi. **Brian E. Dixon, PhD**, Elsevier: Honoraria

